# Effects of exercise training on cardiovascular risk factors in kidney transplant recipients: a systematic review and meta-analysis

**DOI:** 10.1080/0886022X.2019.1611602

**Published:** 2019-05-20

**Authors:** Gang Chen, Liu Gao, Xuemei Li

**Affiliations:** a Department of Nephrology, Peking Union Medical College Hospital, Peking Union Medical College, Chinese Academy of Medical Science, Beijing, China;; b Department of Endocrinology, The Third Hospital of Hebei Medical University, Key Orthopaedic Biomechanics Laboratory of Hebei Province, Shijiazhuang, China

**Keywords:** Exercise, kidney transplant, cardiovascular disease, arterial stiffness, meta-analysis

## Abstract

**Background:** Whether exercise can improve cardiovascular health in kidney transplant recipients (KTRs) is unclear. Therefore, we performed a systematic review of the effects of exercise on cardiovascular risk factors in this population setting.

**Methods:** Randomized control trials (RCTs) evaluating the impact of exercise on major clinical outcomes in KTRs were identified by searches in Cochrane CENTRAL, PubMed, EMBASE, OVID and CBM updated to December 2018. The main outcomes of interest were blood pressure, lipid profile, blood glucose level, arterial stiffness, kidney function, body weight, body mass index, exercise tolerance (VO_2_ peak) and quality of life (QOL).

**Results:** After screening 445 studies in the database, we included 12 RCTs in the review and 11 RCTs for further qualitative analysis. The results indicate a significant improvement in small arterial stiffness [mean difference (MD): −1.14, 95% confidence interval (CI): −2.19–0.08, *p* = .03], VO_2_ peak (MD: 2.25, 95% CI: 0.54–3.69, *p* = .01), and QOL (MD: 12.87, 95% CI: 6.80–18.94, *p* < .01) after exercise intervention in KTRs. However, there is no evidence for an improvement in blood pressure, lipid profile, blood glucose level, kidney function, body weight or body mass index.

**Conclusion:** Exercise intervention in KTRs improves arterial stiffness but does not consistently contribute to the modification of other CVD risk factors like hypertension, dyslipidemia, hyperglycemia, decreased kidney function and obesity. Exercise also improves exercise tolerance and QOL in KTRs.

## Introduction

Kidney transplantation (KTx) is the most desired renal replacement therapy for patients with end-stage renal disease (ESRD), with salutary effects on quality of life (QOL) [1] and overall survival [2] compared to dialysis. However, cardiovascular disease (CVD) remains one of the leading causes of death in kidney transplant recipients (KTRs), accounting for 17% [3] of total deaths, and KTRs have an overall mortality rate at least 5–10-fold greater than the general population [4].

Metabolic syndrome is highly prevalent after KTx, and preexistent comorbidities like hypertension, diabetes and hyperlipidemia continue to be well-recognized contributors to CVD in these patients. Further, the common immunosuppressive choices after KTx also contribute to the burden of CVD risk factors: corticosteroids tend to promote metabolic syndrome due to their antimetabolic effects; cyclosporine is associated with hypertension and hyperlipidemia while tacrolimus is related with insulin resistance and posttransplant diabetes [5].

Exercise is an attractive option for addressing many of the underlying CVD risk factors in KTRs. However, KTRs are at risk for reduced physical fitness due to physical limitation [6], medical comorbid conditions [6–8], skeletal muscle atrophy [8], depression [8], fatigue [6] and lack of motivation [7]. Although the cardiovascular benefits of physical activity in the general population have been confirmed in many studies, only a paucity of studies with conflicting results has been performed among KTRs [9–11]. Thus, it is unclear whether exercise is an effective approach to reducing the risk of CVD in KTRs, and KTRs remain a neglected population concerning exercise recommendations in the 2012 KDIGO (Kidney Disease: Improving Global Outcomes) guidelines [12].

The first meta-analysis of exercise in solid organ transplant recipients, published in 2013, only included 2 randomized control trials (RCT) for KTx [4]. Several newer trials [11,13–18] have been subsequently reported. The solitary meta-analysis on exercise efficacy in KTRs [19] focused only on publications from PubMed and Ichushi, a Japanese medical database; further, this analysis focused on the impact of exercise on exercise performance and QOL, without a specific examination of other CVD risk factors or components of the metabolic syndrome. Here, we undertook a systematic review and meta-analysis of all RCTs with the goal to capture all the available evidence examining exercise and KTx to establish the influence and effect size of various forms of regular exercise training on major clinical outcomes associated with cardiovascular health.

## Methods

### Data sources and selection criteria

The PRISMA (Preferred Reporting Items for Systematic Reviews and Meta-analyses) statement was followed in the conduct of this study [20]. We combined the results from searches of the Cochrane Central Register of Controlled Trials (CENTRAL; from start to December 2018), PubMed (from start to December 2018), EMBASE (from 1980 to December 2018), OVID (from 1993 to December 2018), and China Biology Medicine (CBM, from 1978 to December 2018). For this study, we used the following terms in the search strategy: exercise, physical activity, physical exercise, aerobic exercise, exercise training, isometric exercise, acute exercise, exercise therapy, physical exertion, exercise movement techniques, sports, physical fitness, kidney transplantation, renal transplantation, kidney grafting, and controlled clinical trial (for more details on the search strategy, please see Supplement S-1).

We included prospective RCTs that evaluated the efficacy of regular exercise training on various clinical outcomes in KTRs compared with controls without exercise.

### Data extraction and quality assessment

Data from included studies were scrutinized and extracted by both authors (GC and LG) independently. The Cochrane Data Extraction and Assessment Form were used to explore the research eligibility and decide whether to include the study. Study quality was judged by selection bias, detection bias, attrition bias and completeness of follow-up. Any disagreement about the extracted data and quality assessment was reevaluated by the third author (Dr. Xuemei Li).

### Statistical analysis

According to Cochrane handbook 5-1, the quantitative evaluation was based on the standardized differences between mean values in the intervention and control groups by the Inverse-variance method using RevMan 5.3 (Cochrane, Copenhagen, Denmark). We converted the data in different studies into international units for further meta-analysis. If clinical outcomes were measured more than once in a study, we selected the data closer in time between different studies. We used the model of random effects in RevMan after we assessed the clinical and methodological quality of the RCTs and considered there was heterogeneity among the different studies (Supplement S-2). A *p* value <.05 was considered statistically significant.

Heterogeneity between RCTs was analyzed by Q test of *n* – 1 *df* and *p* < .05 was considered statistically significant. The *I^2^* parameter was used to quantify any inconsistency.

## Results

### Search results

**Figure 1.**
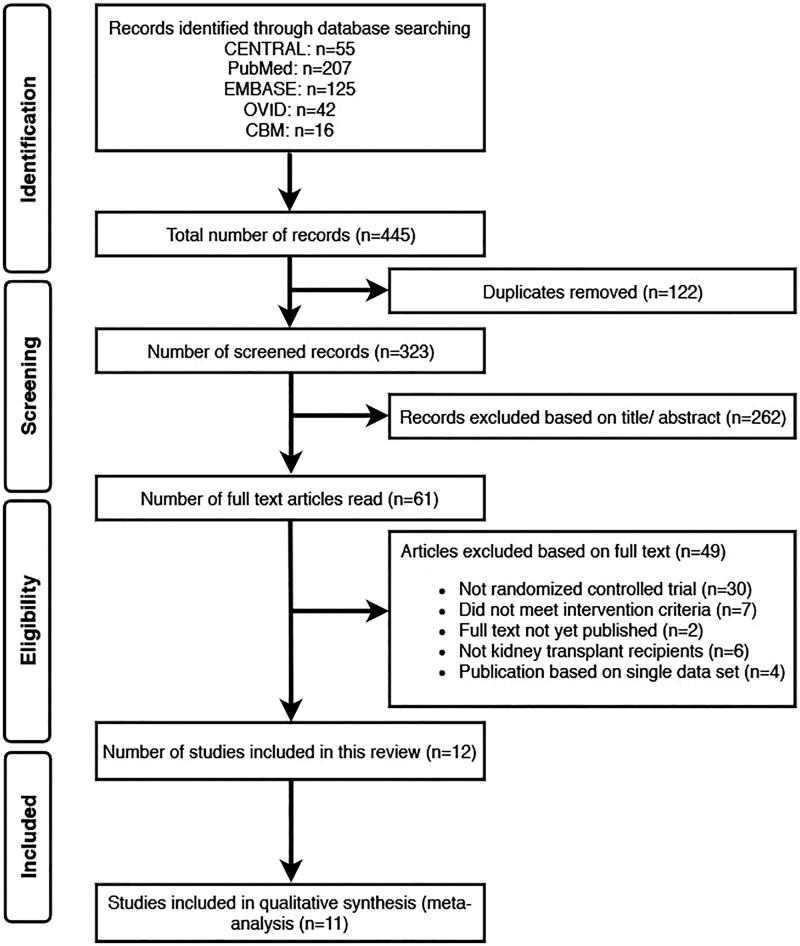
Process of studies selection.

We extracted a total of 445 research papers from CENTRAL (*n* = 55), PubMed (*n* = 207), EMBASE (*n* = 125), OVID (*n* = 42) and CBM (*n* = 16) during the first screen through the databases. One-hundred twenty-two duplications were filtered, and 262 more studies were excluded after review of their titles and abstracts. The two authors (GC and LG) independently inspected the full-text of the 49 remaining references and agreed on including 12 RCTs in this study (Figure 1). The characteristics of the included studies are shown in Table 1. For further qualitative analysis, we extracted data from 11 RCTs and excluded 1 study [17] as it only measured bone mineral density as the primary outcome. Of the 11 RCTs for meta-analysis, aerobic training alone was performed in 2 trials [10,21], resistance training monotherapy was performed in 2 trials [14,16], 6 trials combined aerobic and resistance methods [11,13,15,18,23,24], and 1 trial did not specify the exercise type [22]. For exercise intensity, most trials adopted more than 60% maximum heart rate or maximal oxygen uptake, but 3 studies did not report details on exercise intensity [14,18,22]. Most trials provided a 30–60 min exercise session for 2–4 times per week. In terms of the total intervention period, most trials ranged from 10 weeks to 6 months. Only 1 trial designed the intervention for 4–5 weeks [22] while 3 trials extended the duration to 12 months [10,14,21]. For the controlled arms, patients maintained usual care.

**Table 1.** Characteristics of included studies.

Studies	Country	Mean age (SD), years	Number, *n*	Exercise intervention	Duration	Intensity of intervention	Control	Outcome measurements	Primary outcomes
Painter et al. [21]	US	Ex: 39.7 ± 12.6 (12.6) Con: 43.7 (10.7)	Ex: 54 Con: 43	Home-based exercise (walking or cycling) of at least 30 min per section, 4 times per week	12 months	Initially 60–65% of maximal HR, gradually increased to 75–80% of maximal HR	Routine care	VO_2_ peak, quadriceps muscle strength, body composition, QOL	Exercise results in higher levels of measured and self-reported physical functioning but does not affect body composition.
Painter et al. [10]	US	Ex: 39.7 (12.6) Con: 43.7 (10.6)	Ex: 51 Con: 45	Home-based exercise (walking or cycling) of at least 30 min per section, 4 times per week	12 months	Initially 60–65% of maximal heart rate, gradually increased to 75–80% of maximal HR	Routine care	lipids, BP, maximum METs, BMI	Exercise alone does not reduce CVD risk during the first year after transplantation.
Juskowa et al. [22]	Poland	Ex: 43.75 (12.2) Con: 46.11 (12.3)	Ex: 32 Con: 37	Tailored exercises 15–30 min every other day assisted by physiotherapists	4–5 weeks	No data	Routine care	Biochemical markers of atherosclerosis, muscle strength, pulmonary function	Exercise after KTx produces no significant effects on the markers of atherosclerosis
Min et al. [23]	China	Ex: 35.8 (13.5) Con: 36.1 (12.9)	Ex: 28 Con: 25	Treadmill and resistance training of 45–60 min per section, 3 times a week	6 months	70–85% of maximal HR	Routine care	QOL	Exercise has beneficial effects on the life quality of KTRs.
Kouidi et al. [24]	Greece	Ex: 52.1 (5.6) Con: 52.6 (5.4)	Ex: 11 Con: 12	30–40 min aerobic exercise followed by 10–30 min of strengthening exercises. 4 times a week	6 months	50–75% VO_2_ peak or 65–85% maximal HR	Routine care	VO_2_ peak, tilt test for the evaluation of baroreflex, 24-h Holter for heart rate variability	Exercise increases cardiorespiratory fitness
Pooranfar et al. [13]	Iran	No data	Ex: 29 Con: 15	A combination of aerobic and resistance exercises 60–90 min sessions per week	10 weeks	40–70% maximum HR and resistive exercise with 45%–65% of maximum frequency	Routine care	Sleep quality, lipid profiles	Exercise improves the quality and quantity of sleep; ameliorate lipid profile.
Riess et al. [11]	Canada	Ex: 56.9 (12.2) Con: 52.4 (14.3)	Ex: 16 Con: 15	Endurance (3 days/ week) and strength training (2 days/ week) for 30–60 min per session.	12 weeks	60–80% VO_2_ peak; maintain Borg score 11–13	Routine care	VO_2_ peak, leg strength, 24-hour blood pressure, arterial compliance, QOL, CVD risk score	Exercise improves peak exercise aerobic capacity and cardiac output, muscle strength and QOL.
Tzvetanov et al. [14]	US	Ex: 46 (6.9) Con: 45 (19)	Ex: 9 Con: 8	Individual physical training using low-impact, low-repetition, resistance-based weight training for 1-hour, 2/week.	12 months	No data	Routine care	Body composition, PWV, IMT, weight lifting capacity, GFR, lipid profiles, glucose, QOL	Exercise can improve body composition, kidney function, quality of life.
Greenwood et al. [15]	UK	Ex: aerobic training 53.9 (10.7), resistance training 54.6 (10.6) Con: 49.5 (10.6)	Ex: aerobic training 13, resistance training 13. Con: 20	Aerobic: a tailored training program for 60 min session, 3 times per week. Resistance: 60 min muscle training, 3 times a week.	12 weeks	Borg score 13–15	Routine care	VO_2_ peak, PWV, quadriceps muscle strength, sit-to-stand 60 tests, inflammatory biomarkers, kidney function, QOL	Exercise improves PWV, VO_2_ peak, and muscle strength.
Karelis et al. [16]	Canada	Ex: 45.3 (14) Con: 39.4 (8)	Ex: 10 Con: 10	Individual total body resistance training of 45–60 min, 3 times per week	16 weeks	80% of the 1-repetition maximum	Routine care	VO_2_ peak, muscle strength, BP, lipid profile, OGTT, body composition, cardiometabolic risk factors, and QOL	Exercise training improves strength and QOL, but no change in CVD risk factors.
O’Connor et al. [18]	UK	Ex: aerobic training 53.9 (10.7), resistance training 54.6 (10.6) Con: 49.5 (10.6)	Ex: aerobic training 13, resistance training 13; Con: 20	Twice-weekly supervised, and once-weekly home-based, individually tailored exercise training for 12 weeks. Self-managed exercise continued after that.	9 months	No data	Routine care	PWV, VO_2_ peak, blood pressure, and body weight	Resistance exercise improves PWV. Aerobic exercise improves VO2 peak. No difference in blood pressure or body weight between groups.
Eatemadololama et al. [17]	Iran	Ex: 27.4 (17.36) Con: 36.0 (4.35)	Ex: 12 Con: 12	Supervised upper and lower body resistance training of 80 min, twice per week	12 weeks	Initially 50% of one repetition maximum, and increase 5–10% for the next sessions	Routine care	BMD	Exercise improves long bone mineral density.

Ex: Exercise; Con: Control; US: United States; UK: United Kingdom; HR: Heart rate; BP: Blood pressure; VO_2_ peak: Maximal oxygen uptake; QOL: Quality of life; MET: Maximum exercise tolerance; BMI: Body mass index; BMD: Bone mineral density; CVD: Cardiovascular disease; KTx: Kidney transplantation; KTRs: Kidney transplant recipients; PWV: Pulmonary wave velocity; IMT: Intima-media thickness; GFR: glomerular filtration rate; OGTT: Oral glucose tolerance test.

Borg score: the Borg rating of perceived exertion scale applied to estimate exercise intensity [25].

### Risk of bias in included trials

All participants were randomly assigned in the included trials. The methods of randomization were reported in 7/12 (58.3%) of the trials. Allocation concealment was adequate in 6/12 (50.0%) but unclear in the other 50%. Masked outcome assessment was only done in 2/12 (16.7%) of the studies. Blinded administration in these RCTs was not feasible and none of the participants were blinded to the interventions in any of the studies. The intervention compliance was evaluated in 6/12 (50%) of the trials. Only 2/12 (16.7%) of the trials used intention-to-treat analysis. The detailed clinical and methodological quality assessment of individual trials is available in supplementary (Supplement S-2).

### Study outcomes

We aimed to evaluate the blood pressure (BP), lipid profile, blood glucose level, arterial stiffness, kidney function, body weight and body mass index (BMI), all important indicators of cardiovascular health. The exercise tolerance (VO_2_ peak) and QOL were also included in quantitative analysis.

### Blood pressure

**Figure 2.**
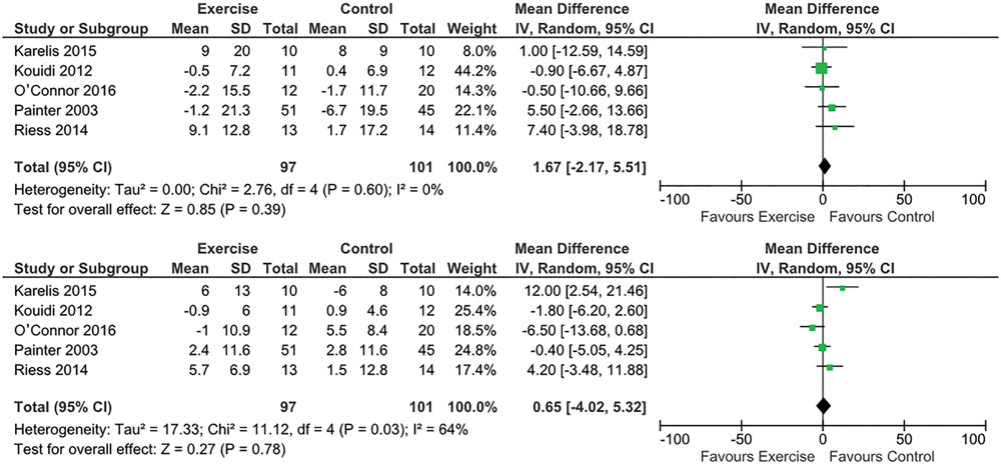
Forest plot of the effects of exercise compared with routine care on systolic BP (upper) and diastolic BP (lower) in KTRs.

Five trials investigated a total of 199 participants to judge the effects of exercise on BP [10,11,16,18,24], using different types of exercise training. Both the systolic and diastolic BP were measured during resting. Data showed that exercise had no effects on either systolic [mean difference (MD) 1.67; 95% confidence interval (CI): −2.17–5.51; *p* = .39] or diastolic BP (MD: 0.65, 95% CI: −4.02–5.32, *p* = .78) (Figure 2).

### Lipid profile

**Figure 3.**
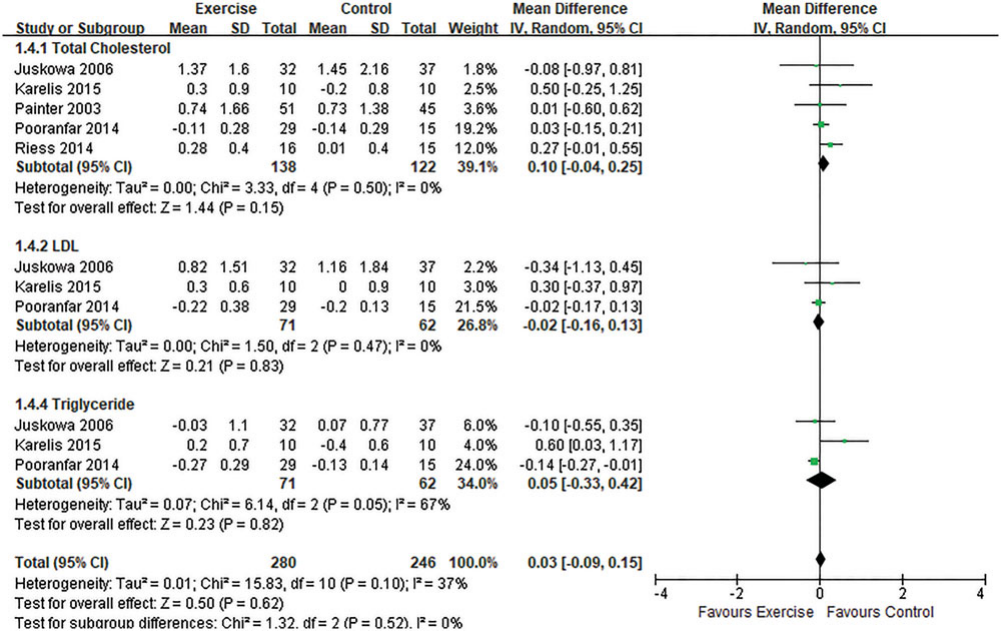
Forest plot of the effects of exercise compared with routine care on the changes in lipid profiles for KTRs.

Three RCTs [13,16,22] assessed lipid profiles including total cholesterol (TC), low-density lipoprotein (LDL) cholesterol, and triglyceride (TG) levels and an additional 2 trials [10,11] examined only TC changes. The referred trials represented aerobic training, resistance training or a combination of both. After the qualitative analysis for totally 261 patients, we found no significant benefits in overall lipid profile after exercise intervention (MD: 0.03, 95% CI: −0.09–0.15, *p* = .62). In subgroup analysis, TC (*p* = .15), LDL cholesterol (*p* = .83) and TG (*p* = .82) were not ameliorated by exercise (Figure 3).

### Blood glucose

Two trials have measured blood glucose changes after exercise intervention in KTRs [14,16]. Karelis et al. employed resistance training in the intervention group for 16 weeks and found no significant changes in either fasting glucose or glucose tolerance [16]. Another study found neutral results in fasting glucose after a 12-month intervention, but the author did not provide available data for further interpretation [14]. As a result, qualitative analysis concerning blood glucose was not feasible.

### Arterial stiffness

**Figure 4.**
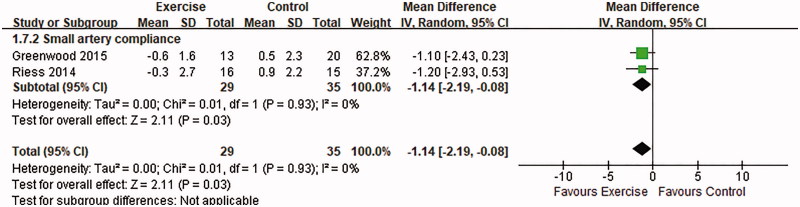
Forest plot of the effects of exercise compared with routine care on changes in small arterial stiffness for KTRs.

Small arterial compliance was examined in 2 trials by either pulmonary wave velocity (PWV) [15] or computerized arterial pulse waveform analysis [11]. Both RCTs measured the clinical outcomes after 12-week intervention. A qualitative analysis including 64 patients showed a consistent improvement in small arterial stiffness after exercise intervention (MD: −1.14, 95% CI: −2.19–0.08, *p* = .03) (Figure 4). In another study, Tzvetanov et al. [14] measured PWV in the intervention group and found a substantial decrease from 9.4 ± 6.3 m/s at baseline to 7.7 ± 1.7 m/s at 12 months, but no data could be obtained for the control group. For large artery compliance, Riess et al. [11] reported no significant modification after exercise therapy (*p* = .95).

### Kidney function

**Figure 5.**
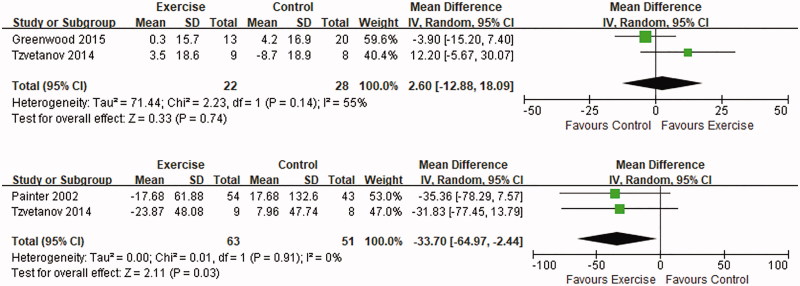
Forest plot of the effects of exercise compared with routine care on the changes in eGFR (upper) and SCr (lower) for KTRs.

To evaluate the impact of exercise on allograft function in KTRs, we included two trials [14,15] which calculated eGFR in a total of 22 subjects in the exercise group and 28 controls. The intervention consisted of 12 months of resistance training [14] or regimens of aerobic or resistance training for 12 weeks [15]. The qualitative evaluation revealed that exercise training was not associated with an improvement in kidney function (MD: 2.60, 95% CI: −12.88–13.09, *p* = .74). Three trials [14,21,22] mentioned serum creatinine (SCr) measurements before and after the intervention, but the baseline SCr in one RCT [22] was obtained before KTx. We included the other 2 trials with baseline SCr after KTx in a meta-analysis and found a significant decrease in SCr after exercise intervention (MD: −33.70, 95% CI: −64.97–2.44, *p* = .03) (Figure 5).

### Body composition

Body weight was measured in 3 RCTs [15,16,18] and BMI was calculated in 4 trials [10,15,16,18]. The intervention consisted of aerobic training, resistance training or the combination, and ranged from 12 weeks to 12 months. The effects of exercise on BW (MD: −2.02, 95% CI: −8.24–4.20, *p* = .52) or BMI (MD: 0.12, 95% CI: −1.52–1.77, *p* = .88) were not significant (Supplement S-3).

### Exercise tolerance

**Figure 6.**
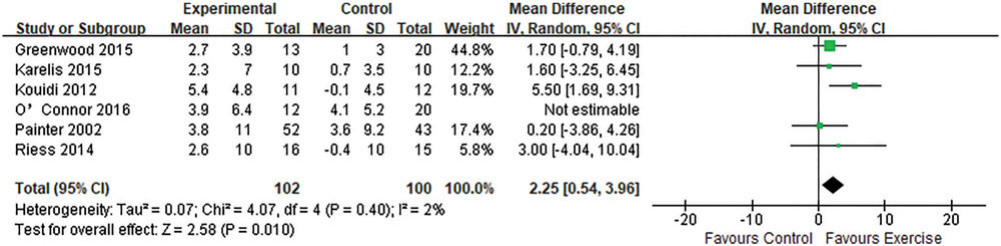
Forest plot of the effects of exercise compared with routine care on the changes in the VO_2_ peak for KTRs.

Six RCTs analyzed VO_2_ peak as an indicator of exercise tolerance [11,15,16,18,21,24]. To generate a qualitative synthesis, we excluded the trial by O’Connor et al. [18] as it was the long-term follow up of the same cohort data reported in Greenwood et al. [15]. The remaining 5 trials had a total of 202 patients and revealed a significant improvement in exercise capacity (MD: 2.25, 95% CI: 0.54–3.69, *p* = .01) after aerobic training [21], resistance training [16] or combined method [11,15,24] over the course of 12 weeks–12 months (Figure 6).

### QOL

QOL was mostly evaluated by the QOL short form (SF)-36 questionnaire in the included RCTs, with assessment of different dimensions like physical functioning [21,23], physical composite score [15,21], physical performance [21,23], social functioning [11,23] and mental health [11,15,23], as well as overall QOL score [11,16]. These trials employed different exercise types for both short and long-term intervention. Generally, exercise improved QOL in different aspects, with significant enhancement in social functioning (MD: 16.76, 95% CI: 2.16–31.37, *p* = .02) and overall QOL scores (MD: 12.87, 95% CI: 6.80–18.94, *p* < .01) (Supplementary S-3).

## Discussion

This is the first quantitative meta-analysis interpreting the effects of exercise intervention on diverse cardiovascular risk factors in KTRs. Although discrepancy existed in exercise type and intervention time of the included studies, we found there was low heterogeneity in the analysis related to systolic BP, lipid profiles, and small arterial stiffness. Results of the present study indicate that exercise tolerance and QOL are significantly enhanced after the exercise intervention. However, the usefulness of exercise in amending traditional CVD risk factors such as hypertension, hyperlipidemia and hyperglycemia is questionable. Encouragingly, the recently recognized CVD risk factor arterial stiffness does appear to improve after exercise in KTRs.

Traditional CVD risk factors identified in population-based cohorts such as the Framingham Heart Study include age, sex, smoking status, systolic BP, TC, high-density lipoprotein cholesterol, left ventricular hypertrophy and diabetes; [26] the combination of these risk factors can be used to predict 10-year-risk for CVD [26]. A more recent version of the Framingham risk prediction strategy includes diastolic BP and LDL cholesterol as additional CVD risk factors [27]. A substantial body of evidence has demonstrated the benefits of exercise on BP [28,29], lipid profiles [30] and blood glucose [31,32] in the general population, highlighting a central role for exercise in the primary and secondary prevention of CVD. However, our meta-analysis shows that the benefit of exercise may not extend to KTRs, at least for several of these traditional risk factors. There are some possible explanations: (1) many traditional CVD risk factors are pathophysiologic determinants rooted in long-term unadjusted life habits and they cannot be easily attenuated with a relatively short-term exercise intervention; (2) the multiple pathological factors that contribute to the high rates of CVD in KTRs are too strong to overcome by the monotherapy of exercise, and may instead require a combinatorial approach with other lifestyle interventions; (3) KTRs still suffer from the residual effects of renal failure while the administration of steroids or calcineurin inhibitors reverses the benefits of exercise; and (4) to best appreciate the impact of exercise and its impact on CVD risk, changes in maintenance medications that might result from exercises, such as a reduction in antihypertensives and lipid-lowering therapy should also be considered. However, there was no extractable data in the included RCTs for this issue. To better design trials to judge the effects of exercise, the dosing and categories of these medications should be considered.

In contrast to a lack of effect on BP, lipid profiles, and fasting glucose, exercise does have a beneficial impact on arterial stiffness, even after intervention as short as 3 months. Arterial stiffness has emerged as an additional CVD risk factor [33,34] and a systematic review shows that the consideration of PWV can improve the prediction of CVD events [35]. Increased age, metabolic syndrome, and inflammation, risk factors enriched in the ESRD population, all contribute to arterial stiffening [36]. Some studies have shown that increased arterial stiffness in patients with chronic kidney disease increases the likelihood of progressive kidney loss and a substantial predisposition to CVD [37]. The qualitative analysis demonstrates that the addition of exercise can improve the artery stiffness in KTRs, in addition to the restore of kidney function.

It has also been recognized that the decreased level of kidney function is an independent CVD risk factor [38,39]. In our analysis, exercise therapy did not improve kidney function as assessed by eGFR. The two trials included in the qualitative analysis provided inconsistent results [14,15]. Greenwood et al. [15] found no improvement in eGFR whereas Tzvetanov et al. [14] found an increase in eGFR and lower SCr in the exercise group compared with control. We noticed that the latter one was a personalized rehabilitation program designed for obese KTRs. The explanations for the discrepancy may be personalized guidance in exercise or that obese patients benefit better in exercise.

Obesity, which exacerbates metabolic syndrome and inflammatory status, is an additional CVD risk factor [40,41]. The unchanged body weight and BMI after exercise intervention are somehow unexpected, especially in the trial targeted at obese KTRs for a 12-month long intervention [14]. However, the KTRs may improve their appetite and gain weight thanks to the depletion of uremic status after transplantation. It is possible that detailed body composition improves with exercise, without a measurable change in body weight or BMI, but data on the hip to waist ratio, %lean weight, etc. were not available for analysis. Alternatively, in the absence of simultaneous dietary guidance, patients might mistakenly adopt a high-calorie or a high-fat diet with increased activity and thus compromise the beneficial effects of exercise.

VO_2_ peak is an established method to determine cardiorespiratory function [15]. In our analysis, we included 3 more RCTs than the only previous meta-analysis of exercise in KTRs [19] and demonstrated a benefit of exercise in KTRs. We also examined more components of the QOL evaluation, again showing a benefit for exercise. Thus, independent of an effect on CVD risk factors, these results underscore a benefit for exercise in overall physical and mental wellbeing.

There are some limitations to our systematic review. First, the RCTs addressing this topic are of heterogeneous quality, and for several of the clinical outcomes of interest, the number of trials eligible for analysis is relatively small. Second, it was not feasible to conduct blinded RCTs by using exercise intervention and the compliance to the intervention was not evaluated in some studies, thus compromising the quality of the trials. Third, the majority of the RCTs included limited participants and conducted an intervention for no more than 6 months. Therefore, to better address the efficacy of exercise on CVD in KTRs, we would recommend further clinical studies to include more participants and to evaluate the long-term effects by using a certain type of exercises that could be easily quantified with the intervention intensity.

## Conclusion

Our systematic review demonstrates that exercise in KTRs has a mixed impact on CVD risk profiles. It improves arterial stiffness, exercise tolerance and QOL, but does not significantly improve other key CVD risk factors such as hypertension, dyslipidemia, hyperglycemia, decreased kidney function and obesity. Additional long-term RCTs examining a greater number of patients are needed to understand the effects of exercise on cardiovascular health in KTRs.

## Supplementary Material

Supplementary file 3

Supplementary file 2

Supplementary file 1
