# Supplementary material for: Effects of exercise training on cardiovascular risk factors in kidney transplant recipients: a systematic review and meta-analysis
Source: Ren Fail. 2019 May 20;41(1):408–18. doi: 10.1080/0886022X.2019.1611602 (PMC6534232; doi:10.1080/0886022X.2019.1611602)
Supplement: Supplementary file 3 [file IRNF_A_1611602_SM0872.docx]

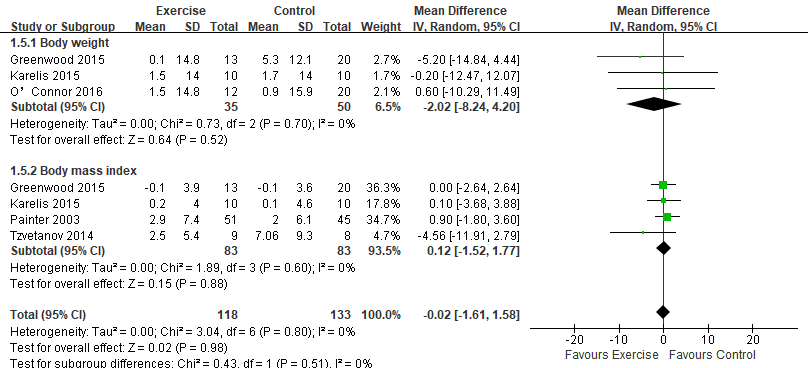


Fig. Forest plot of the effects of exercise compared with routine care on the changes in body composition for KTRs.


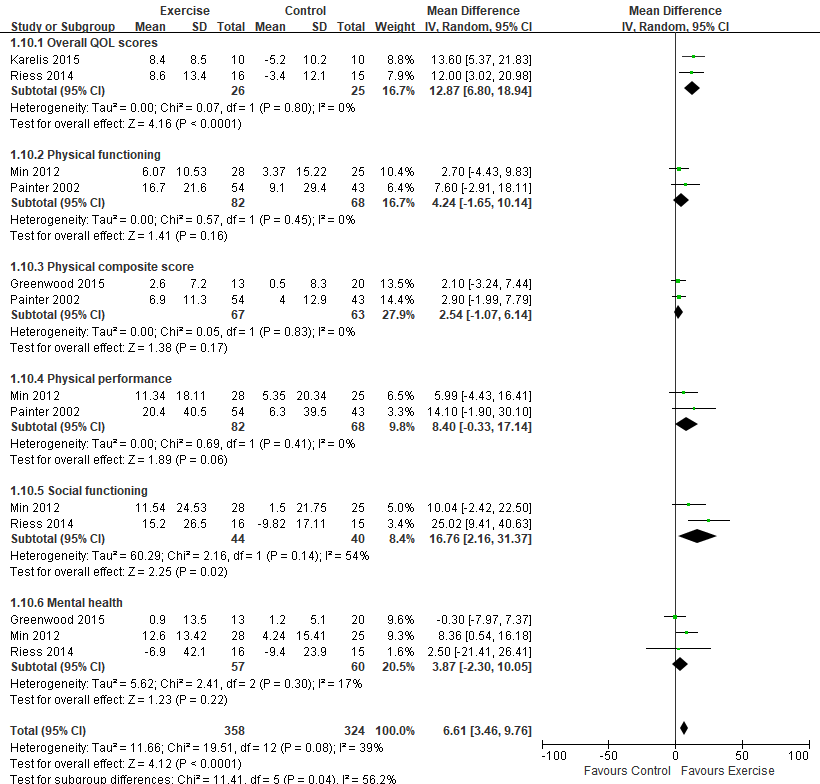


Fig. Forest plot of the effects of exercise compared with routine care on the changes in QOL (quality of life) for KTRs.
