# Supplementary material for: Effects of exercise training on cardiovascular risk factors in kidney transplant recipients: a systematic review and meta-analysis
Source: Ren Fail. 2019 May 20;41(1):408–18. doi: 10.1080/0886022X.2019.1611602 (PMC6534232; doi:10.1080/0886022X.2019.1611602)
Supplement: Supplementary file 2 [file IRNF_A_1611602_SM0871.docx]

**S-2** **Methodological quality assessment of included trials**.

NA= not applicable as it is not possible in physical exercise intervention trials. ● = Fulfilling criteria ○ = Unclear x = not done.

A = Eligible/considered for inclusion, B = Enrolled/randomized, C= Analyzed, D = Per cent followed, E = Percentage of compliance.

|  | Recruitment | Method of randomization | Allocation concealment | Masked outcome assessments | Blinded administration | Blinded participants | Completeness of follow-up | | | | | intention-to-treat analysis |
| --- | --- | --- | --- | --- | --- | --- | --- | --- | --- | --- | --- | --- |
|  |  |  |  |  |  |  | A | B Exp/Ctrl | C Exp/Ctrl | D Exp/Ctrl | E Exp/Ctrl |  |
| Painter 2002 | ● | ● | ● | ○ | NA | X | 257 | 83/84 | 54/43 | 64/51 | 58 | ● |
| Painter 2003 | ● | ● | ● | ○ | NA | X | ○ | 51/45 | 51/45 | 100/100 | ○ | ● |
| Juskowa 2006 | ○ | ○ | ○ | ○ | NA | X | ○ | 32/37 | 32/37 | 100/100 | ○ | X |
| Min 2012 | ○ | ● | ○ | ○ | NA | X | ○ | 28/25 | 28/25 | 100/100 | ○ | X |
| Kouidi 2012 | ● | ● | ● | ○ | NA | X | 33 | 12/12 | 11/12 | 92/100 | 80 | X |
| Pooranfar 2014 | ● | ○ | ○ | ○ | NA | X | ○ | 29/15 | 29/15 | 100/100 | ○ | X |
| Riess 2014 | ● | ● | ● | ○ | NA | X | 113 | 16/15 | 16/15 | 100/100 | 81 | X |
| Tzvetanov 2014 | ○ | ○ | ○ | ○ | NA | X | ○ | 9/8 | 9/8 | 100/25 | 100/25 | X |
| Greenwood 2015 | ● | ● | ● | ● | NA | X | 209 | 40/20 | 26/20 | 65/100 | 87.4 | X |
| Karelis 2015 | ● | ○ | ○ | ○ | NA | X | 102 | 12/12 | 10/10 | 83/83 | 80 | X |
| O’Connor 2016 | ● | ● | ● | ● | NA | X | 209 | 40/20 | 22/20 | 55/100 | ○ | X |
| Eatemadololama 2017 | ● | ○ | ○ | ○ | NA | X | ○ | 12/12 | 12/12 | 100/100 | ○ | X |
