# Supplementary material for: Effects of exercise training on cardiovascular risk factors in kidney transplant recipients: a systematic review and meta-analysis
Source: Ren Fail. 2019 May 20;41(1):408–18. doi: 10.1080/0886022X.2019.1611602 (PMC6534232; doi:10.1080/0886022X.2019.1611602)
Supplement: Supplementary file 1 [file IRNF_A_1611602_SM0870.docx]

**Item S1.** Detailed Search Strategy

CENTRAL

| 1. MeSH descriptor: [Exercise] explode all trees 2. MeSH descriptor: [Sports] explode all trees 3. "physical activity":ti,ab,kw (Word variations have been searched) 4. "physical exercise":ti,ab,kw (Word variations have been searched) 5. Aerobic Exercise:ti,ab,kw (Word variations have been searched) 6. Exercise Training:ti,ab,kw (Word variations have been searched) 7. MeSH descriptor: [Exercise Therapy] explode all trees 8. MeSH descriptor: [Physical Exertion] explode all trees 9. MeSH descriptor: [Physical Fitness] explode all trees 10. MeSH descriptor: [Exercise Movement Techniques] explode all trees 11. #1 or #2 or #3 or #4 or #5 or #6 or #7 or #8 or #9 or #10 12. MeSH descriptor: [Kidney Transplantation] explode all trees 13. "renal transplantation":ti,ab,kw (Word variations have been searched) 14. Kidney Grafting:ti,ab,kw (Word variations have been searched) 15. renal transplantations:ti,ab,kw (Word variations have been searched) 16. #12 or #13 or #14 or #15 17. #11 and #16 |
| --- |

PubMed

1. Search "Exercise"[Mesh]
2. Search Physical Activity
3. Search Activities, Physical
4. Search Activity, Physical
5. Search Physical Activities
6. Search Exercise, Physical
7. Search Exercises, Physical
8. Search Physical Exercise
9. Search Physical Exercises
10. Search Exercise, Aerobic
11. Search Aerobic Exercise
12. Search Aerobic Exercises
13. Search Exercise Training
14. Search Isometric Exercise
15. Search Acute Exercise
16. Search "Exercise Therapy"[Mesh]
17. Search "Physical Exertion"[Mesh]
18. Search "Exercise Movement Techniques"[Mesh]
19. Search "Sports"[Mesh]
20. Search "Physical Fitness"[Mesh]
21. Search (((((((((((((((((((#1) OR #2) OR #3) OR #4) OR #5) OR #6) OR #7) OR #8) OR #9) OR #10) OR #11) OR #12) OR #13) OR #14) OR #15) OR #16) OR #17) OR #18) OR #19) OR #20
22. Search "Kidney Transplantation"[Mesh]
23. Search Renal Transplantation
24. Search Renal Transplantations
25. Search Transplantations, Renal
26. Search Transplantation, Renal
27. Search Grafting, Kidney
28. Search Kidney Grafting
29. Search Transplantation, Kidney
30. Search Kidney Transplantations
31. Search Transplantations, Kidney
32. Search (((((((((#22) OR #23) OR #24) OR #25) OR #26) OR #27) OR #28) OR #29) OR #30) OR #31
33. Search (#21) AND #32
34. Search randomized controlled trial [pt]
35. Search controlled clinical trial [pt]
36. Search randomized [tiab]
37. Search placebo [tiab]
38. Search drug therapy [sh]
39. Search randomly [tiab]
40. Search trial [tiab]
41. Search groups [tiab]
42. Search (((((((#34) OR #35) OR #36) OR #37) OR #38) OR #39) OR #40) OR #41
43. Search (#33) AND #42
44. Search animals [mh] NOT humans [mh]
45. Search (#43) NOT #44
46. Search (#33) AND #45

EMBASE

| 1. exp randomized controlled trial/ 2. exp randomization/ 3. exp double blind procedure/ 4. exp single blind procedure/ 5. random $.tw. 6. or/1-5 7. (animal or animal experiment).sh. 8. human.sh 9. 7 and 8 10. 7 not 9 11. 6 not 10 12. exp clinical trial/ 13. (clin $ adj3 trial $).tw. 14. ((singl $ or doubl $ or trebl $ or tripl $) adj3 (blind $ or mask $).tw. 15. exp placebo/ 16. placebo $.tw. 17. random $.tw. 18. exp experimental design/ 19. exp crossover procedure/ 20. exp control group/ 21. exp latin square design/ 22. or/12-21 23. 22 not 10 24. 23 not 11 25. exp comparative study/ 26. exp evaluation 27. exp prospective study/ 28. (control $ or prospective $ or volunteer $).tw. 29. or/25-28 30. 29 not 10 31. not (11 or 23) 32. 11 or 24 or 31 33. exp "Physical Activity Capacity and Performance"/ 34. exp Kinesiotherapy/ 35. Exercise/ 36. or/33-35 37. exercise.tw. 38. (resistance training or resistance program$).tw. 39. (physical fitness or physical rehabilitation).tw. 40. (strength$ and (muscle or program$ or training)).tw. 41. or/37-40 42. or/36,41 43. exp Kidney Transplantation/ 44. (renal transplant$ or kidney transplant$).tw. 45. or/43,44 46. 36 and 45 and 32 |
| --- |

OVID

1. randomized controlled trial.pt.
2. controlled clinical trial.pt.
3. randomized.ab.
4. placebo.ab.
5. drug therapy.fs.
6. randomly.ab.
7. trial.ab.
8. groups.ab.
9. 1 or 2 or 3 or 4 or 5 or 6 or 7 or 8
10. animals.sh.not (humans.sh. and animals.sh.)
11. 9 not 10
12. Exercise sh.
13. Physical activity
14. Sports
15. Exercise therapy
16. 12 or 13 or 14 or 15
17. Kidney transplantation
18. Renal transplantation
19. 17 or 18
20. 16 and 19 and 11

CBM

1. 锻炼 【扩展全部树】/全部副主题词
2. 运动 【扩展全部树】/全部副主题词
3. 有氧运动
4. 无氧运动
5. #1 or #2 or #3 or #4
6. 肾移植 【扩展全部树】/全部副主题词
7. 肾脏移植
8. #6 or #7
9. #5 and #8
